# Clinical relevance of *Aspergillus *isolation from respiratory tract samples in critically ill patients

**DOI:** 10.1186/cc4823

**Published:** 2006-02-17

**Authors:** Koenraad H Vandewoude, Stijn I Blot, Pieter Depuydt, Dominique Benoit, Werner Temmerman, Francis Colardyn, Dirk Vogelaers

**Affiliations:** 1 Department of Intensive Care, Ghent University Hospital, Ghent, Belgium; 2 Hogeschool Gent, Health Care Department "Vesalius", Ghent, Belgium; 3 Department for Infectious Diseases, Ghent University Hospital, Ghent Belgium

## Abstract

**Introduction:**

The diagnosis of invasive pulmonary aspergillosis, according to the criteria as defined by the European Organisation for the Research and Treatment of Cancer/Mycoses Study Group (EORTC/MSG), is difficult to establish in critically ill patients. The aim of this study is to address the clinical significance of isolation of *Aspergillus *spp. from lower respiratory tract samples in critically ill patients on the basis of medical and radiological files using an adapted diagnostic algorithm to discriminate proven and probable invasive pulmonary aspergillosis from *Aspergillus *colonisation.

**Methods:**

Using a historical cohort (January 1997 to December 2003), all critically ill patients with respiratory tract samples positive for *Aspergillus *were studied. In comparison to the EORTC/MSG criteria, a different appreciation was given to radiological features and microbiological data, including semiquantitative cultures and direct microscopic examination of broncho-alveolar lavage samples.

**Results:**

Over a 7 year period, 172 patients were identified with a positive culture. Of these, 83 patients were classified as invasive aspergillosis. In 50 of these patients (60%), no high risk predisposing conditions (neutropenia, hematologic cancer and stem cell or bone marrow transplantation) were found. Typical radiological imaging (halo and air-crescent sign) occurred in only 5% of patients. In 26 patients, histological examination either by ante-mortem lung biopsy (*n *= 10) or necropsy (*n *= 16) was performed, allowing a rough estimation of the predictive value of the diagnostic algorithm. In all patients with histology, all cases of clinical probable pulmonary aspergillosis were confirmed (*n *= 17). Conversely, all cases classified as colonisation had negative histology (*n *= 9).

**Conclusion:**

A respiratory tract sample positive for *Aspergillus *spp. in the critically ill should always prompt further diagnostic assessment, even in the absence of the typical hematological and immunological host risk factors. In a minority of patients, the value of the clinical diagnostic algorithm was confirmed by histological findings, supporting its predictive value. The proposed diagnostic algorithm needs prospective validation.

## Introduction

*Aspergillus *is a saprophytic filamentous fungus widespread in the environment. Although *Aspergillus *can affect any organ system, the respiratory tract is involved in more than 90% of affected patients. Inhalation of *Aspergillus *spores or conidia can give rise to various clinical conditions, depending essentially on the host's immunological status [1,2]. In immunocompetent patients, pulmonary aspergilloma, allergic bronchopulmonary aspergillosis and obstructive bronchial aspergillosis are described. In immunocompromised patients, especially with prolonged neutropenia, *Aspergillus fumigatus *can invade the pulmonary parenchyma, resulting in invasive pulmonary aspergillosis, a disease with a high lethality. More recently, a locally invasive form called necrotizing pulmonary aspergillosis has been described in patients with mild immunosuppression [1,3-5]. Recent data indicate that invasive aspergillosis must be considered as an emerging and devastating infectious disease in intensive care unit (ICU) patients even in the absence of an apparent predisposing immunodeficiency. In a carefully designed study in a medical ICU, the incidence of invasive aspergillosis was 5.8% ; the majority of these patients did not have a history of hematological malignancy. [6]. In an autopsy study of ICU patients, 2.7% of patients were found to have invasive aspergillosis. Chronic obstructive pulmonary disease (COPD) and advanced liver cirrhosis were recognised as potential risk factors [7].

The significance of isolation of *Aspergillus *from respiratory cultures has been studied extensively in immunocompromised hosts who develop invasive pulmonary aspergillosis [8-10]. On the other hand, little is known about the significance of isolation of *Aspergillus *from respiratory specimens of apparently immunocompetent or mildly immunocompromised patients. Because species of *Aspergillus *are ubiquitous, one must be cautious in ascribing a pathogenic role to the fungus obtained from a nonsterile site. Therefore, diagnosis of invasive pulmonary aspergillosis on the basis of an *Aspergillus *positive culture from tracheal aspirates remains most difficult in patients with intermediate risk [5], or in patients without currently recognized risk factors. The golden standard for the definite diagnosis of proven invasive pulmonary aspergillosis remains histopathological lung tissue examination. In clinical practice, the diagnosis of proven invasive pulmonary aspergillosis is rarely established ante-mortem, because of the critical condition of the patients, excluding invasive procedures. Since no non-invasive diagnostic test is sensitive or specific enough to establish definite diagnosis, the diagnostic categories of 'probable' and 'possible invasive pulmonary aspergillosis' have been developed, based on the combination of host risk factors, clinical symptoms and distinct radiological and microbiological criteria [11]. These diagnostic criteria were originally developed for clinical trials in patients with bone marrow transplants and cancer. However, in ICU patients, clinical signs and symptoms are often non-specific, and except for neutropenia and a congenital or acquired immunocompromised state, it is not feasible to define particular host risk factors, or combinations of risk factors, for the acquisition of invasive fungal disease, since there are no large epidemiological studies in this special patient population.

The aim of the present study is to assess the clinical relevance of *Aspergillus *positive respiratory tract samples in ICU patients, based upon a diagnostic algorithm derived from the European Organisation for the Research and Treatment of Cancer/Mycosis Study Group (EORTC/MSG) criteria for invasive fungal disease [11] with a modified interpretation of medical imaging data and microbiological findings. The validity of the diagnostic criteria was assessed if biopsy or necropsy data were available.

## Materials and methods

### Setting

The present study was conducted in the Ghent University Hospital, a 1,060 bed primary care and referral centre with a 54 bed ICU including a surgical and medical ICU, an ICU for cardiac surgery and a unit for severely burned patients. Approximately 3,800 patients are admitted to the ICU each year. The surgical ICU serves all kinds of surgery with the need for intensive care management, including multiple trauma and solid organ transplantations. During the study period, 910 patients received a solid organ transplant (kidney, pancreas, liver and heart).

The medical ICU serves all patients with internal diseases requiring intensive care, including patients with haematological malignancies and bone marrow transplant recipients; a total of 270 haematological patients was admitted during the study period. For immunocompromised patients or patients colonized or infected with epidemiologically important microorganisms, each unit is equipped with several isolation rooms. The burns unit consists of six separated isolation rooms with shower and bath installations within.

### Study design

The study is designed as a historical cohort study (retrospective analysis of prospectively gathered data), including all patients admitted to the ICUs during the period January 1997 through December 2003. The sole criterion for entry in the study is a lower respiratory tract culture positive for *Aspergillus *spp. As a routine practice, all intubated patients in the ICU receive surveillance cultures of endotracheal aspirate thrice weekly. Otherwise, respiratory specimens from all patients, including pulmonary biopsy and specimens of normally sterile sites, are obtained according to the instructions of the attending physicians. The local Center for Hospital Hygiene and Infection Control prospectively files all patient records with any positive culture for *Aspergillus *spp., hence all relevant data could be retrieved.

Patients admitted to the ICU with prior diagnosis of invasive *Aspergillus *disease were not included in the analysis.

Data collection and processing, and patient anonymisation were done according to legal regulations and local Ethics Committee requirements. Given the non-interventional design, the Ethics Committee of the Ghent University Hospital waived informed consent.

### Definitions of definite or probable invasive pulmonary aspergillosis and *Aspergillus *colonisation

An adapted clinical algorithm considering clinical status, host factors, microbiological data, bronchoscopy with broncho-alveolar lavage, medical imaging and cytological examination of smears of broncho-alveolar lavage fluid results was used to discriminate colonisation from invasive infection. These criteria for defining cases of invasive pulmonary aspergillosis are summarized in Table 1. For the diagnosis of probable invasive pulmonary aspergillosis, all criteria needed to be fulfilled (1 + 2 + 3 + either 4a or 4b). This algorithm is in part derived from the EORTC/MSG consensus data concerning opportunistic invasive fungal infections in immunocompromised patients with cancer and hematopoetic stem cell transplants [11]. The circulating galactomannan test for *Aspergillus *antigen was not routinely available in our institution during the study period, and was hence not taken into the diagnostic elaboration. Patients not fullfilling the criteria for invasive pulmonary aspergillosis were classified as colonized. Autopsy was performed at the request of the attending physician after consent of the family.

### Data collection

The following data relevant to patient characteristics were collected: age, Acute Physiology and Chronic Health Evaluation (APACHE) II score [12], comorbidities and underlying diseases, and treatment with systemic and inhalation corticosteroids. Data collected concerning ICU treatment and outcome were ICU stay, ventilator dependence, need for vasopressor or inotropic treatment, need for renal replacement therapy, and antifungal therapy. Outcome was described as in-hospital mortality, defined as death within the same hospital episode as the ICU admission.

### Classification of radiological findings

Results of chest X-ray and thoracic CT scan were described as normal, acute respiratory distress syndrome (ARDS)-like, non-specific infiltrates and consolidation, pleural fluid, nodular lesion(s), halo sign, air-crescent sign, and cavitation. The CT halo sign is described as a mass-like infiltrate with a surrounding halo of ground glass attenuation. The halo lesion was shown to correspond to a central fungal nodule surrounded by a rim of hemorrhage and coagulative necrosis. The air-crescent sign is described as a pulmonary cavitation [13,14].

### Other definitions

Acute renal failure is defined as the need for renal replacement therapy, acute respiratory failure as the need for acute mechanical ventilation and cardiovascular failure as the need for inotropic or vasopressive support despite adequate fluid resuscitation [15-18].

### Statistics

Continuous variables are described as median (interquartile range). Comparative analyses were performed with the Mann-Whitney *U* or Chi-square test when appropriate. Survival curves were prepared by means of the Kaplan-Meier method and univariate survival distributions were compared with use of the Log rank test. Statistical analyses were performed with SPSS 11.0 (SPSS Inc., Chicago, IL, USA). All used tests are two-tailed and statistical significance is defined as *P *< 0.05.

## Results

**Figure 1:**
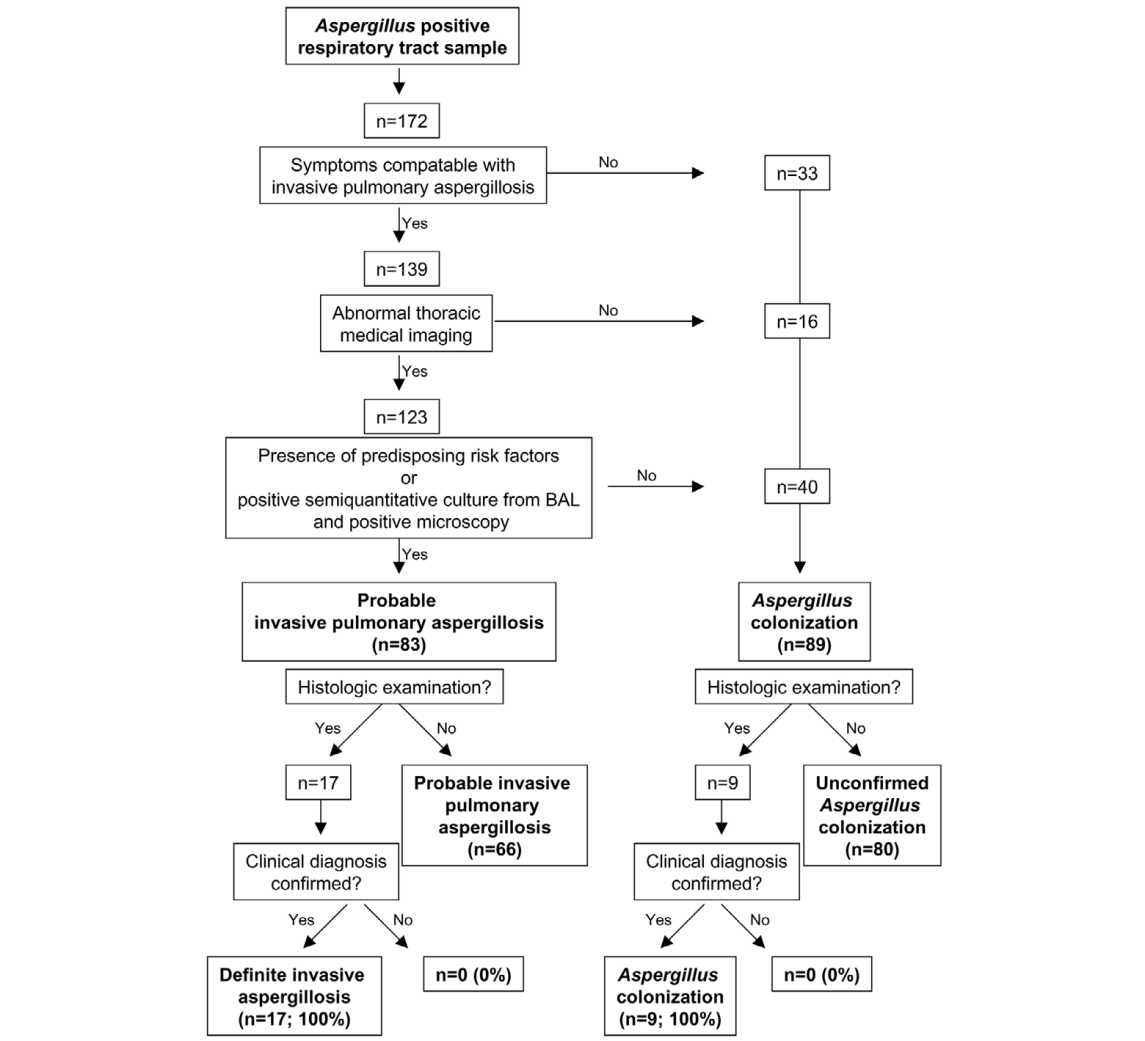
Diagnostic breakdown of the study cohort (172 patients).

During the observation period, 25,216 patients were admitted to the ICU. Respiratory tract samples were positive for *Aspergillus *in 172 patients (incidence: 6.8/1,000 ICU admissions). The diagnostic breakdown of the cohort is illustrated in Figure 1. According to the predefined criteria, 83 cases (48.3%) were classified as invasive pulmonary aspergillosis (17 definite, 68 probable). In the remaining 89 patients (51.7%), the presence of *Aspergillus *was considered as colonisation. Pulmonary biopsy was performed in ten patients. Biopsy was positive in seven patients, who were classified as documented invasive aspergillosis ante-mortem. In three patients, classified clinically as colonisation, lung biopsy showed no fungal disease. Autopsy in patients with an *Aspergillus *positive respiratory tract specimen was performed in 16 patients. Ten of these patients fullfilled the predefined criteria of probable invasive pulmonary aspergillus ante-mortem; since lung necropsy specimens confirmed the diagnosis, they were subsequently classified as definite invasive pulmonary aspergillosis. In six patients who were considered as colonized ante-mortem, the autopsy did not reveal invasive *Aspergillus *disease.

In Table 2, underlying conditions of patients with invasive pulmonary aspergillosis and colonisation are summarized. Of the patients diagnosed with invasive pulmonary aspergillosis, 40% of patients had a high risk profile (neutropenia, hematological cancer, bone marrow or stem cell transplant). Patient characteristics and outcome are summarised in Table 3. Thoracic medical imaging (Table 4) shows that nodular lesions were almost exclusively found in invasive pulmonary aspergillosis (30% versus 2%; *P *< 0.001). The halo and air-crescent sign were evident in only three patients. Most patients classified as invasive pulmonary aspergillosis had non-specific radiological findings.

**Figure 2:**
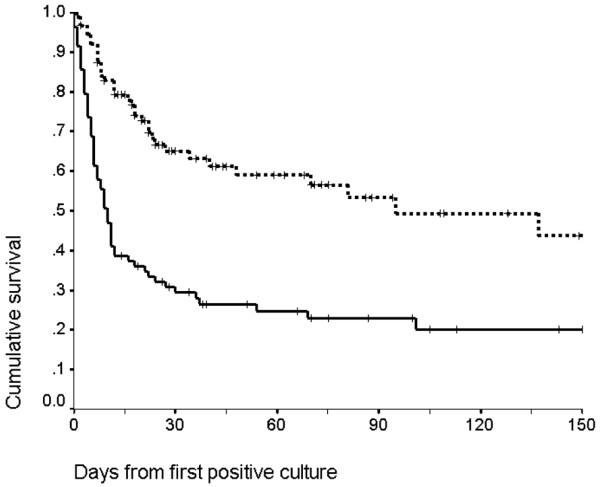
Survival curves for intensive care unit patients with invasive pulmonary aspergillosis and *Aspergillus *colonisation (Log rank test: *P *< 0.001). Patients with invasive aspergillosis are represented by the solid line; patients with *Aspergillus *colonisation are represented by the dashed line.

Appropriate antifungal treatment was given to 71 (85.5%) patients with invasive pulmonary aspergillosis. All patients classified with invasive pulmonary aspergillosis in whom no antifungal therapy was started died (*n* = 12). When these patients were excluded, the mortality rate was 73%. Figure 2 shows the survival curves of patients categorised as invasive aspergillosis and colonisation.

## Discussion

Until recently, research on epidemiology and risk factors for the acquisition of *Aspergillus *infection and treatment of invasive disease has almost enterily focused on severely immunocompromised patients with hematological malignancy and solid organ recipients. However, recent literature indicates an expanding spectrum of patients at risk for invasive aspergillus disease. These are categorised into high risk (allogeneic bone marrow transplant, neutropenia and hematological cancer), intermediate risk (autologous bone marrow transplant, malnutrition, corticosteroids, HIV, solid organ transplant, diabetes, underlying pulmonary disease and solid organ cancer) and low risk (cystic fibrosis and connective tissue disease) [5]. Furthermore, case reports and papers about invasive pulmonary aspergillosis in COPD patients and apparently non-immunocompromized patients [19-26] have been published.

Hence, it seems worthwile to address the question of diagnosis of invasive pulmonary aspergillosis in ICU patients. The lack of validated and stringent criteria for case definitions in patient categories, other than hemato-oncological and solid organ transplant, hampers diagnostic assessment. The ante-mortem diagnosis of proven invasive aspergillosis is extremely difficult to establish in ICU patients as hemodynamic and/or respiratory insufficiency and coagulopathy often preclude invasive tissue sampling. Because of these diagnostic limitations, a feasible diagnostic approach was developed. As in the EORTC/MSG definitions, host factors for the acquisition of invasive disease were taken into account. For patients who did not meet the criteria for high-risk host, the *Aspergillus *spp. positive tracheal aspirate had to be corroborated with a positive semi-quantitative culture and a positive cytological examination of broncho-alveolar lavage fluid. This is in part endorsed by the observations of Greub and Bille [27] in a case-definition study in immunocompromised patients: compared to those from patients considered to be colonised, cultures of lower respiratory tract specimens from patients with proven invasive pulmonary aspergillosis showed a significant difference in the total number of *Aspergillus *colonies recovered from culture per episode; for BAL (broncho-alveolar lavage), the number of *Aspergillus *colonies per agar plate was also significantly higher in the proven aspergillosis group. Furthermore, many authors consider the visualisation of the characteristic septate hyphae in bronchial washings as a confirmatory finding of invasive disease in the presence of a compatible clinical picture [8,28-32]. False-positive results appear to be unusual, since patients without chronic lung diseases rarely show colonisation of the lower tracheobronchial tree with *Aspergillus *[33]. Compared to the EORTC/MSG diagnostic criteria, the interpretation of radiological data in the algorithm is also less strict, as any major radiological sign of pneumonia is taken into consideration. Medical imaging of the thorax in ICU patients is less pathognomonic due to many confounding factors such as ventilator associated pneumonia, atelectasis, and pleural fluid effusions in critically ill ventilated patients; furthermore, it can be speculated that typical radiological lesions may be less apparent because of the difference in severity and nature of the immune derangements. Typical lesions for invasive aspergillosis, such as the halo and the air-crescent sign, were only found in 5% of patients. This is in agreement with the low sensitivity of 24% in patients without hematological malignancy compared with 82% in patients with neutropenic hematological malignancy [34].

Since the modified clinical diagnosis of probable invasive aspergillosis is less stringent than the EORTC/MSG criteria, a lower specificity may be of concern. However, in a limited number of patients, histopathological specimens were available in order to check the validity of the clinical assumption. Samples for histology were available in 26 patients. Of these, 13 fullfilled the EORTC/MSG criteria for host risk factors. Ten patients underwent pulmonary biopsy. Seven of these met the criteria of probable aspergillosis prior to biopsy, and could be reclassified ante-mortem as definite invasive pulmonary aspergillosis because of a positive histopathological examination. In three other patients, classified as colonized, lung biopsy showed no evidence of fungal infection. Furthermore, autopsy data were available in 16 patients, of whom 10 were classified as probable invasive pulmonary aspergillosis ante-mortem, and the other six patients as colonized. Necropsy findings histologically confirmed the clinical diagnosis in all these patients. These data are in support of a high positive predictive value of the criteria for the diagnosis of invasive pulmonary aspergillosis. Nevertheless, the number of patients with histological confirmation was low. The true predictive value of the proposed diagnostic algorithm needs to be assessed prospectively.

In this study, using an entry criterion of an *Aspergillus *positive respiratory specimen and an adapted diagnostic algorithm, an incidence of invasive pulmonary aspergillosis of 3.2/1,000 ICU admissions was found. In the subgroup of medical patients, the incidence was three times as high (10.2/1,000). In a recent retrospective study in a medical ICU, an incidence of invasive aspergillosis of even 5.8% was found, with, in most cases, pulmonary involvement [6]. In a study considering autopsies of patients from a mixed medical-surgical ICU, an incidence of 2.7% of proven invasive aspergilosis was found [7]. In general, the autopsy rate is low in our institution (<5%) because of local ethical regulations. It can not be excluded that patients classified as colonized indeed had invasive disease, and that other patients with negative surveillance cultures suffered from invasive disease since respiratory tract cultures lack sensitivity. It is clear that a stringent protocol for post-mortem examination is necessary for a truthful estimation of the epidemiology and incidence of invasive aspergillosis in ICU patients [6,7]. Furthermore, prospective research should include non-culture methods for diagnosis, such as the detection of galactomannan, PCR, and beta-D-glucan in non-neutropenic patients [35,36]. At this time, it is unclear if these non-invasive methods are of any diagnostic value in critically ill patients without the EORTC/MSG host risk factors.

An important finding is that the majority of the patients classified as having invasive pulmonary aspergillosis did not belong to the well known high-risk group (neutropenia, bone marrow transplant, hematological cancer); this is in accordance with the data provided in the study by Meersseman and colleagues [6]. Underlying conditions, such as COPD, chronic lung disease, non-hematological malignancy, HIV infection, diabetes mellitus, liver failure, chronic alcohol abuse, malnutrition and extensive burns, have been described in association with invasive aspergillosis [5,7,37,38]. In the EORTC/MSG diagnostic criteria, the use of corticosteroids for more than three weeks is considered a predisposing host factor [11]. In the setting of persistent septic shock, steroids are frequently used since a beneficial effect has been demonstrated [39]. Corticosteroids substantially impair macrophage killing of *Aspergillus *spores and mononuclear cell killing of *Aspergillus *hyphae [40]. In the setting of underlying lung disease, there is a risk factor for invasive aspergillosis at much lower doses and shorter courses of steroids [41,42]. This should be taken into account in the clinical assessment of an *Aspergillus *spp. positive respiratory tract sample. Steroid treatment has also been given important weight in a recently described point-score system for assessment of positive cultures [43]. It has been speculated that patients with normal immune function prior to ICU admission may be at risk for invasive aspergillosis due to a temporary immunoparalysis in the context of the multiple organ dysfunction syndrome [44].

Hospital mortality of patients with invasive pulmonary aspergillosis in this study was high (77%), but in accordance with previous reports describing dramatic fatality rates [22,24,45,46]. When comparing the survival curves of the group of patients with invasive aspergillosis with the group of patients classified as colonised, a clear difference is observed during the first 15 days after positive respiratory *Aspergillus *culture and fullfillment of the diagnostic criteria for invasive disease. The initial decline of the curve of patients with invasive disease is more pronounced, reflecting an acute mortality probably due to *Aspergillus *infection. This fits well with the generally accepted time frame of the development of invasive *Aspergillus *disease until demise. This observation is an indirect argument in favour of the value of the diagnostic algorithm.

## Conclusion

The finding of an *Aspergillus *positive respiratory tract sample in an ICU patient cannot be discarded and must trigger further diagnostic exploration using BAL, with semiquantitative culture and cytological examination, as well as CT scan and pulmonary biopsy if possible. Adapted clinical diagnostic criteria should be used in order not to miss a critical window of therapeutic opportunity.

The proposed diagnostic algorithm for the diagnosis of invasive pulmonary aspergillosis is supported by histopathological data from a subgroup of patients. An important finding is that not only patients with severe hematological disease are afflicted: the majority of patients has an intermediate risk for the acquisition of invasive disease. Radiological features are often non-specific. The associated mortality is high in spite of appropriate treatment.

## Key messages

• 	The finding of *Aspergillus *spp. in respiratory tract samples in critically ill patients should not be routinely discarded as colonisation, even in presumably immunocompetent hosts.

• 	Clinical signs and symptoms of invasive pulmonary aspergillosis and radiographic features are often non-specific in ICU patients

• 	Risk factors for the development of invasive aspergillosis in critically ill patients include neutropenia, haematological malignancy and immunosuppressive treatment. However, invasive disease can occur in the absence of these risk factors.

• 	In the presence of clinical features of unresolving pneumonia, appropriate antifungal therapy should be considered carefully when *Aspergillus *spp. is isolated from respiratory tract specimens, in patients with COPD, after corticosteroid exposure even in moderate dose, and in other patients with severe underlying disease and critical illness induced immunoparalysis.

## Abbreviations

COPD = chronic obstructive pulmonary disease; EORTC/MSG = European Organisation for the Research and Treatment of Cancer/Mycoses Study Group; ICU = intensive care unit.

## Competing interests

The authors declare that they have no competing interests.

## Authors' contributions

KV, WT and DV conceived and designed the study. Acquisition of the data was performed by KV and WT. Statistical analysis was performed by SB and PD. Interpretation of the results was done by KV, SB, DV, FC and PD. KV and SB drafted the manuscript, after which it was revised by DV, PD, FC and DB.

## Figures and Tables

**Table 1:** Criteria for defining cases of invasive pulmonary aspergillosis

Definite invasive pulmonary aspergillosis
A. Positive result of histological testing and positive result of culture from lung tissue obtained by biopsy or autopsy
B. Positive result of culture of a specimen obtained from a normally sterile site by use of aseptic invasive technique
Probable invasive pulmonary aspergillosis
1. *Aspergillus*-positive lower respiratory tract specimen culture
2. Compatible signs and symptoms
Fever refractory to at least three days of appropriate antibiotic therapy
Recrudescent fever after a period of defervescence of at least 48 hours while still on antibiotics and without other apparent cause
Pleuritic chest pain
Pleuritic rub
Dyspnoea
Hemoptysis
Worsening respiratory insufficiency in spite of appropriate antibiotic therapy and ventilatory support
3. Abnormal medical imaging by portable chest X-ray or computerised tomography of the lungs
4. Either
a. Host risk factors: one of the following conditions
Neutropenia (absolute neutrophil count less then 500/mm^3^) preceding or at the time of ICU admission
Underlying haematological or oncological malignancy treated with cytotoxic agents
Glucocorticoid treatment (prednisone or equivalent, >20 mg/day)
Congenital or acquired immunodeficiency
Or
b. Semiquantitative *Aspergillus*-positive culture of BAL (+ or ++), without bacterial growth together with a positive cytological smear showing branching hyphae
*Aspergillus *colonisation
Not fullfulling the criteria for proven or probable invasive pulmonary aspergillosis

ICU, intensive care unit; BAL, broncho-alveolar lavage.

**Table 2:** Underlying diseases in intensive care unit patients with respiratory tract samples positive for *Aspergillus *spp.

Underlying condition	Associated with invasive aspergillosis^a^	Associated with *Aspergillus *colonisation
Hematological malignancy^b^	28 (2)	3
With neutropenia	6 (1)	1
Post bone marrow/stem cell transplantation	2	0
Myelodysplastic syndrome	4	*-*
Solid tumor with chemotherapy	3	1
Solid tumor without chemotherapy	5	3
Graves' disease	-	1
Immunosuppressive therapy	14 (4)	8
Solid organ transplant	8 (2)	4
Auto-immune disease	4 (2)	4
Lung fibrosis	2	0
Aplastic anemia	1	1
Chronic obstructive pulmonary disease	29 (4)	25
Requiring chronic systemic corticosteroid use	21 (3)	12
Requiring inhalation corticosteroids	24 (3)	16
Asthma	2	1
Liver cirrhosis	3 (2)	2
Malnutrition	3	-
Diabetes mellitus	8 (1)	9
Alcoholism	5 (2)	3
Chronic heart failure	6	5
Chronic renal failure – dialysis dependent	3 (1)	2
Lung fibrosis	2	1
Active Cytomegalovirus disease	3	1
Active tuberculosis	-	1
Absence of known underlying disease	14 (5)	27

^a^Numbers in parentheses indicate cases with definite invasive aspergillosis. ^b^*P* value < 0.05 for difference between patients with invasive aspergillosis (definite + probable) and colonisation.

**Table 3:** Patient characteristics for intensive care unit patients with invasive pulmonary aspergillosis and *Aspergillus *colonisation

Characteristic	Definite invasive aspergillosis (*n* = 17)	Probable invasive aspergillosis (*n* = 66)	Invasive aspergillosis (*n* = 83)	*Aspergillus *colonisation (*n* = 89)	*P*^a^
Age (years)	54 (51–63)	61 (50–69)	61 (51–69)	66 (47–72)	0.186
APACHE II score	28 (21–30)	28 (20–31)	28 (19–34)	24 (20–29)	0.021
Neutropenia	3 (17.7)	8 (12.1)	11 (13.3)	2 (2.2)	0.008
Hemodynamic instability	17 (100)	49 (74.2)	74 (89.2)	58 (65.2)	< 0.001
Acute renal failure	10 (58.8)	23 (34.9)	33 (39.8)	23 (25.8)	0.073
Acute respiratory failure	17 (100)	63 (95.5)	80 (96.4)	77 (86.5)	0.029
Duration of mechanical ventilation prior to first positive culture (days)	2 (1–8)	4 (1–9)	3 (1–8)	3 (1–7.5)	0.690
Duration of mechanical ventilation (days)	11 (6–24)	15 (6–32)	13 (5–29)	9 (3–21)	0.031
Length of ICU stay prior to first positive culture (days)	2 (1–8)	4 (1–9)	4 (1–8)	5 (1–9)	0.396
Length of ICU stay (days)	11 (7–24)	15 (7–34)	14 (7–31)	13 (6–25)	0.383
Number of cultures positive for *Aspergillus *spp.	2 (2–3.5)	2 (2–3)	2 (2–3)	1 (1–2)	< 0.001
Number of patients examined with BAL	12 (70.6)	49 (74.2)	61 (73.5)	29 (32.6)	< 0.001
28-days mortality	15 (88.2)	44 (66.7)	59 (49.0)	26 (23.1)	< 0.001
In-hospital mortality	16 (94.1)	48 (72.7)	64 (77.1)	36 (40.4)	< 0.001

Continuous variables are described as median (interquartile range); categorical variables are described as *n* (%). ^a^*P* value for invasive aspergillosis (*n* = 83) versus *Aspergillus *colonisation (*n* = 89). No significant differences were observed between patients with proven versus probable invasive aspergillosis. APACHE, Acute Physiology and Chronic Health Evaluation; ICU, intensive care unit; BAL, broncho-alveolar lavage

**Table 4:** Radiological findings in intensive care unit patients with invasive pulmonary aspergillosis or *Aspergillus *colonisation

Radiological finding	Invasive aspergillosis (*n* = 83)^a^	*Aspergillus *colonisation (*n* = 89)
Normal^b^	0	30
Diffuse reticular or alveolar opacities (ARDS like)^b^	12 (1)	4
Non-specific infiltrates and consolidation	42 (10)	49
Pleural fluid	0	5
Nodular lesions^b^	25 (5)	1
Air-crescent sign	1	0
Halo sign	2 (1)	0
Cavitation	1	0

^a^Numbers in parentheses indicate cases with definite invasive aspergillosis. ^b^*P* value < 0.05 for differences between the groups. ARDS, acute respiratory distress syndrome.
